# Detection of Material Degradation of a Composite Cylinder Using Mode Shapes and Convolutional Neural Networks

**DOI:** 10.3390/ma14216686

**Published:** 2021-11-06

**Authors:** Bartosz Miller, Leonard Ziemiański

**Affiliations:** Faculty of Civil and Environmental Engineering and Architecture, Rzeszów University of Technology, al. Powstańców Warszawy 12, 35-959 Rzeszów, Poland; ziele@prz.edu.pl

**Keywords:** shell, layered composites, mode shapes, non-destructive tests, machine learning

## Abstract

This paper presents a numerical study of the feasibility of using vibration mode shapes to identify material degradation in composite structures. The considered structure is a multilayer composite cylinder, while the material degradation zone is, for simplicity, considered a square section of the lateral surface of the cylinder. The material degradation zone size and location along the cylinder axis are identified using a deep learning approach (convolutional neural networks, CNNs, are applied) on the basis of previously identified vibration mode shapes. The different numbers and combinations of identified mode shapes used to assess the damaged zone size and location were analyzed in detail. The final selection of mode shapes considered in the identification procedure yielded high accuracy in the identification of the degradation zone.

## 1. Introduction

Composite structures are increasingly used in various applications, including responsible and distinct fields such as aviation [1,2,3], or mechanical and civil engineering [4,5]. Therefore, any damage or degradation of material properties may lead to very dangerous situations, not only threatening the significant financial losses but also primarily endangering the lives and health of passengers of aircraft in which composite structures are used or users of composite building structures.

Regular inspections of the condition of such responsible structures are necessary by the regulations of relevant institutions (e.g., in Poland, the Civil Aviation Authority [6] for aviation or the General Directorate for National Roads and Motorways [7] for motorways and bridges). The process of “in situ, nondestructive sensing and analysis of structural characteristics, including the structural response, to estimate the severity of damage/deterioration and evaluate the consequences thereof on the structure in terms of response, capacity, and service-life” (see [8]) is called Structural Health Monitoring (SHM). A group of so-called Non-Destructive Techniques (NDTs) used in SHM gathers methods that “examine materials and structures without impairment of serviceability and reveals hidden properties and discontinuities, or differences in characteristics without destroying the serviceability of the part or system” (see [9]); in the case that no failures or defects are identified, the tested item can be used again for service. Non-destructive techniques are broadly applied in composite structures testing; some examples of NDTs are provided below:

- *Visual inspection* is the oldest and the most popular method of structure testing. In its classic version it does not require any specialized equipment; only the naked eye of a trained and experienced specialist is necessary [3,10]. Nowadays, it is sometimes allied not as a standalone method but as an aid to other kinds of instrumented NDTs [11], often supported by advanced optical equipment and modern image-processing methods (e.g., deep learning [12]);
- *Ultrasonic testing*: this method uses ultrasonic waves mechanically induced by a transducer converting an electric signal into mechanical one; through the analysis of the wave transmission or reflection, it is able to detect, e.g., delamination or inclusions [1,11]; ultrasonic testing is described as “the most versatile of the testing techniques available to industry” [13];
- *Thermography*: this method is based on the observation of heat distribution over the tested sample’s surface and the search for hot spots that may indicate a defect in the component/object under examination; the test object may be heated by mechanical force, by illumination with a very strong light, by a laser, or by any other method [10,14,15,16];
- *Radiographic testing*: this technique is similar to well-known medical radiography; it uses X-radiation (electromagnetic radiation with energy within 10–50 kV); X-rays passing through the specimen are recorded on an X-ray-sensitive medium, and the analysis of the recorded radiographic image makes it possible “to examine composites for flaws and compliance to manufacturing standards” [17,18,19].

There are, of course, several other widely recognized methods (e.g., liquid penetrant testing, electromagnetic tests, acoustic emission tests, guided waves, penetrating tests, eddy current tests, terahertz spectroscopy, vibrotermography, or shearography); there are also some new tests being developed (e.g., guided waves with contactless excitation [20] or self-heating-based vibrothermography [21]).

Each of the listed methods has its advantages and disadvantages; some are useful for testing the entire structure (e.g., vibration tests); others are designed for local testing (e.g., ultrasonic testing). With the development of measurement techniques, new opportunities arise and new techniques for nondestructive structural testing and damage detection are developed.

This paper focuses on vibration-based structural damage identification. The idea behind vibration-based methods is that any structure’s change (including damage) affects its mass, stiffness, and damping. That, in turn, causes changes of such dynamic parameters as natural frequencies, mode shapes, and modal damping. The analysis of the dynamic parameters can be carried out in three different domains: time, frequency, and time-frequency domains, where the majority of approaches recently fall into the frequency domain (see [22]).

The natural frequencies-based methods analyze the changes of natural frequencies. The natural frequencies are easy to measure using a limited number of sensors, but their changes due to occurring damage are usually small [23]. Moreover, the problem of identifying structural changes based on natural frequencies changes is often ill-posed [23]. Natural frequencies-based methods are also insensitive to local damage [22]. Like frequency-based methods, methods based on modal damping are not widely used today, mainly due to the complexity of the problem and the necessity of selecting an appropriate damping model [24].

Among the vibration-based damage identification approaches, the most commonly used are those based on mode shapes. The application of mode shapes is justified by the great sensitivity of the mode shapes to local damage and lesser sensitivity to environmental effects such as temperature change [25]. The analyzed quantities may be modal shapes changes caused by the appearance of damage. The assumption that the modal vectors vary near damage leads to detection of damage presence and its identification. However, the accuracy of damage identification improves dramatically when instead of direct model shape changes, the curvature or strain mode shapes are analyzed [26,27,28]. Some modern signal processing methods are also applied, especially on experimental data when the measurements of the original structure (before damage appears) are not available [23]. Among these methods are, e.g., fractal dimension method [29], wavelet transform method [30] or Hilbert-Huang transform [31]. However, the mode shape-based approach lacks attention to the problem noted and analyzed in optimization problems: mode shapes crossing and mode shape identification, that is, the designation of the natural frequency and the corresponding vibration mode shape with a suitable “mode shape identifier” (see [32,33,34,35,36]).

This paper is a proposal of a non-destructive technique using a modern full-field measuring approach (e.g., Digital Image Correlation (DIC) [37,38] or scanning laser Doppler vibrometry [39]) in monitoring composite structures through the measurement and analysis of vibration mode shapes. The mode shapes, after identification, are used as input data to the procedure of material degradation zone identification. The identification of the material degradation is performed by artificial neural networks following the principle of *deep learning*, namely, convolutional neural networks (CNNs) [40,41,42]. The convolutional network is a specialized kind of neural network designed for advanced data processing [43], widely applied in computer vision and pattern-recognition problems, object detection, speech recognition, biomedical systems, and natural language processing. The popularity and wide range of applications of CNNs are due to the following advantages: (i) CNNs combine feature extraction and feature classification processes into a single learning procedure; they can learn features of the model in the training phase directly from input data; (ii) because CNNs’ neurons are weakly connected with associated weights, CNNs can process large input data with high computational efficiency compared to conventional Multi-Layer Perceptron (MLP) networks; (iii) CNNs are robust to small input data transformations, including translation, scaling, skewing, and distortion; (iv) CNNs can adapt to different input data sizes. Due to these advantages, convolutional neural networks are now widely used with great success in numerous practical applications for their strong local feature extraction capability and flexible architectures and have thus become the standard for recognition systems and image or video processing [44]. In recent years, CNNs have also been readily used in SHM systems, mainly for vibration analysis [45,46,47,48].

The main difference between a classical neural network (now called a shallow neural network) and a convolutional network is the fact that a shallow network uses—as the main operator—general multiplication, whereas a CNN uses convolution (i.e., an operation on two functions that produces a third function); the convolution operation is conducted on the local receptive field to extract local features. In convolutional network terminology, arguments to the convolution are often referred to as the input and the kernel, whereas the output is referred to as the feature map. Unlike classical neural networks built from flat layers, a CNN is built from 3D sections, where neurons are ordered in three dimensions, which allows for feature detection in the image as well as in time series [49,50]. CNN processing capabilities have been used repeatedly in applications related to computational mechanics [51], vibration analysis [44], and SHM [45,46,48].

As shown in [52], CNNs have better generalization and recognition abilities than shallow networks trained using back-propagation (BP) principle but need more computational power for learning. At this time, however, this drawback of CNNs is not crucial. The advantages of CNNs are even more obvious when image analysis is necessary, as shown, e.g., in [53,54,55], the performance of a CNN is superior to both shallow networks and support vector machines (SVMs).

In this paper, a new, successfully developed approach for the identification of the material degradation zone of multilayer composite structures is discussed. To overcome the limitations of the traditional approaches (2D analysis, high spatial resolution of mode shapes description), CNNs are applied to create a tool to analyze the mode shapes of vibrations and to draw conclusions about the appearance, location, and size of the material degradation zone in the analyzed structure. This paper is a continuation of the research of the same authors (see [36]), where the CNN-based identification of mode shapes is presented. Herein, on the basis of identified mode shapes, the location and size of the material degradation zone are assessed. The CNN-based procedure is accurate and effective.

The presented work is the first attempt, known to the authors of this paper, to use previously identified vibration mode shapes as an exclusive source of information during the identification of damage in a composite structure.

The paper is organized as follows: Section 2 contains the formulation of the problem. Section 3, the main section of the article, discusses the CNN-based identification of a material degradation zone. Section 4 contains the discussion of the results. The conclusions and future research directions are reported in Section 5.

## 2. Formulation of the Problem

### 2.1. Solution of the Vibration Problem

The equation of motion for linear dynamic is governed by the generalized equation of dynamic equilibrium [56]:

(1) $$M\ddot{x}+C\dot{x}+Kx=P\;\;,$$

where the matrices M, C, and K represent the mass, damping, and stiffness of the structure, respectively. The vector x represents nodal displacements, and P represents time-dependent external forces. *Dot notation* is used to denote the derivatives, namely, $\dot{x}$ and $\ddot{x}$ are the first and the second derivatives of x with respect to time *t*, respectively.

When P and C are neglected (this means that external forces are constant over time or can be ignored and damping is treated as negligibly small), Equation (1) is simplified to:

(2) $$M\ddot{x}+Kx=0\;\;.$$

This approach leads to the generalized eigenproblem [57]:

(3) $$K\Phi =M\Phi \Omega^{2},\;\;\;$$

where the Φ matrix gathers the eigenvectors $\phi_{i}$, and each of them describes one mode shape corresponding to subsequent angular frequency contained in the diagonal matrix Ω. The angular frequencies divided by 2π give the natural frequencies $f_{i}$:

(4) $$f_{i}=\frac{\omega_{i}}{2\pi }\;\;.$$

Each of the $\phi_{i}$ eigenvectors (mode shapes) describes the maximal deformation of the structure when it vibrates harmonically with a corresponding $f_{i}$ resonant frequency.

### 2.2. Investigated Structure and Its Finite Element Model

The structure analyzed in this paper is a thin-walled composite cylinder, fixed at one end (displacement degrees of freedom fully locked) and free at the other end. The total length of the cylinder is 6.0 m; the radius of the shell middle surface is 0.6103 m. The thickness of the shell is 0.016 m; the number of layers of composite material is 4, 8, 16, or 32. Material parameters are as follows: Young’s moduli $E_{1}=141.9$ GPa, $E_{2}=9.78$ GPa, Poisson’s ratio $\nu_{12}=0.42$, shear modulus $G_{12}=6.13$ GPa, and density ρ=1445 kg/m${}^{3}$; they correspond to the graphite–epoxy composite material often referred to as a benchmark material in numerical simulations; see, e.g., [58].

**Figure 1:**
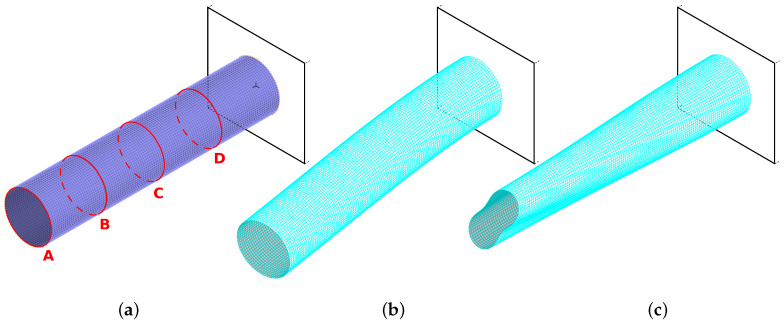
The finite element (FE) model (**a**) and two examples of vibration mode shapes: (**b**) B11, (**c**) C21.

The FEM model used a regular FE mesh (see Figure 1a), the number of columns of elements parallel to the cylinder symmetry axis was fixed at 80, and the number of layers was also fixed at 120. The FE model had a total of 9680 nodes and 58,000 degrees of freedom. The multilayer finite element applied in the model (called MITC4 in Adina FE code, [59]) was a four-node element, following first-order shear theory assumptions.

All the calculation have been performed using Adina FE code; the choice of finite element and FE mesh density was based on the experience gained by the authors in previous research (see [34,35,60,61]), where FE convergence was verified and the results from different FEM codes were compared. Neither higher FE mesh density nor the change of an FE element to a higher order one caused a significant changes in the analyzed quantities, and in particular, do not affect the mode shapes investigated in this paper.

### 2.3. Convolutional Neural Networks

A CNN is a hierarchical structure, stacking multiple layers, such as the convolution layers (for feature extraction), batch normalization layers (regularization effect: the training of network is much faster), pooling layers (down-sampled feature map for dimension reduction), and activation layers (activate nonlinear mapping—rectified linear units, ReLU).

**Figure 2:**
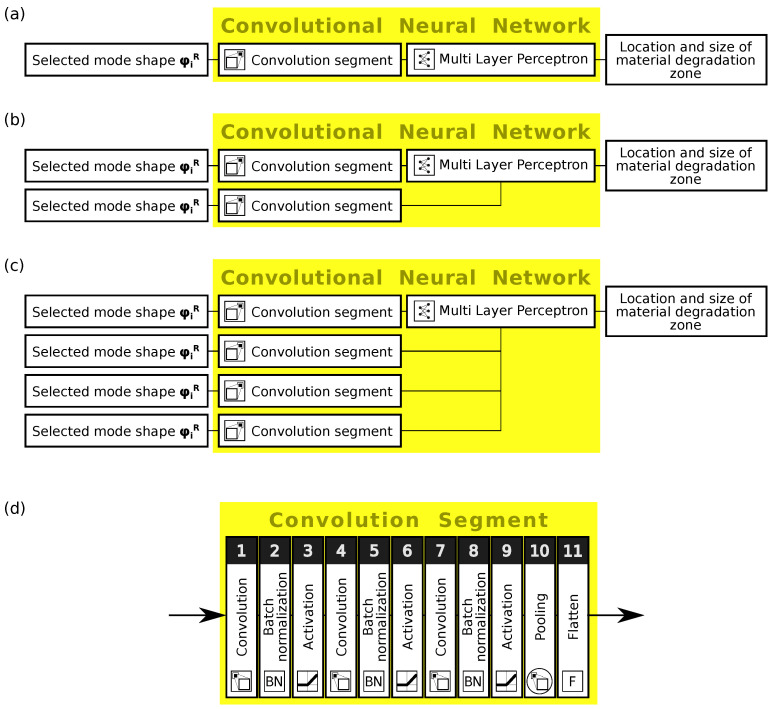
The scheme of CNN applied for the identification of material degradation zone: (**a**) one mode shape as input, (**b**) two mode shapes, (**c**) four mode shapes, and (**d**) convolution segment.

In the paper, a novel CNN is constructed to automatically learn features from the input data (mode shapes). The CNN is used as a regression network applied to identify a location and size of a zone of local degradation of material properties using one or more mode shapes (three-dimensional matrix of nodal displacements) as a source of input data. CNNs are trained using the RMSProp algorithm [43]. A learning set consisted of 3000 patterns; 2400 were selected randomly and moved to the teaching set, the remaining 600 patterns created the validation set. The number of learning epochs depended on continuously analyzed results obtained for validation patterns: an increase in the validation error during a certain number of epochs marked the end of learning. The architecture of CNNs applied here is summarized in Table 1 and shown in Figure 2, where Figure 2d shows the main part of the networks called herein the *convolution segment*.

All simulations using CNNs were performed in the commercial code Mathematica (V12.0, Wolfram Research Inc., Champaign, IL, USA) environment [62].

## 3. Identification of Material Degradation Size and Location

### 3.1. Material Degradation Zone

**Figure 3:**
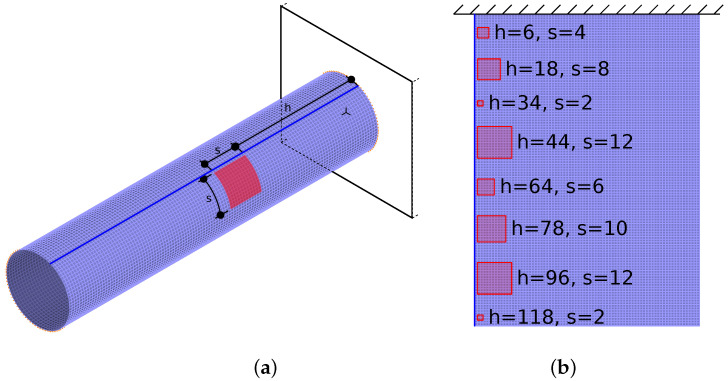
The cylinder (**a**) and its lateral surface unrolled (**b**), with some examples of material degradation zones and the corresponding values of parameters *h* (location) and *s* (size).

In a randomly selected “square” area (see Figure 3a), the material constants of half of the shell layers are significantly reduced, namely, Young’s moduli have values of $E_{1}=14.18$ GPa and $E_{2}=0.978$ GPa in these layers, instead of original values $E_{1}=141.8$ GPa, $E_{2}=9.78$ GPa. This area is in what is called the *material degradation zone*. Figure 3a shows an example of a material degradation zone location; Figure 3b shows the unrolled lateral surface of the cylinder, with eight examples of different degradation zones (all cases appear separately; cases with the appearance of several degradation zones simultaneously were not considered).

The considered locations of the degradation zone along the circumferential direction of the cylinder have the left border aligned to the same column of finite elements in the FE model; the right border is calculated according to the randomly selected size *s* of the degradation zone. The location in the axial direction depends on the random *h* parameter (describing the row of finite elements calculated from the built-in end of the cylinder). The considered values of *s* are 2, 4, 6, 8, 10, and 12 (in terms of finite elements’ rows and columns); for *h*, the considered values change from 2 to 119 with the step of 1; to analyze the accuracy of the *h* identification, depending on the distance from the built-in end of the cylinder, the values of *h* are—where necessary—divided into 10 classes, each with the size of 12 elements and centers in elements 12(c−1)+6 (where *c* is the number of the class). Although the parameters *s* and *h* are originally described by the number of finite elements, in what follows they are expressed in meters (m). The length of the finite element side along the cylinder’s axial direction equals 50 mm and along circumferential direction 47.9 mm; therefore, for the calculation of *h* in meters, the element size 50 mm is considered, for *s*—47.9 mm. The parameter values expressed in meters are as follows: s={0.0959,0.1917,0.2876,0.3835,0.4793,0.5751} m, h∈{0.1,5.95} m with the step of 0.05 m.

Some examples of the location *h* and size *s* conversion from the number of elements (NbFE, see Figure 3) to length units (here in millimeters) are shown in Table 2.

### 3.2. The Degradation Zone Identification Procedure

**Figure 4:**
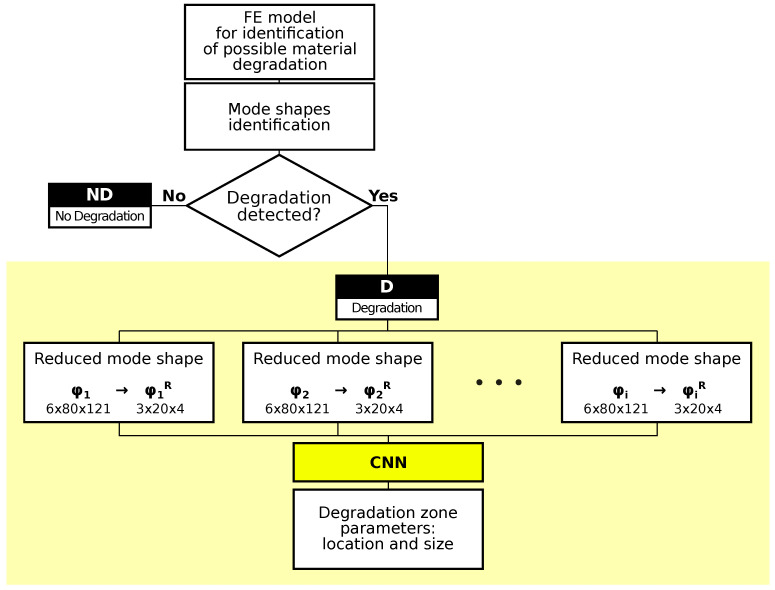
The scheme of the material degradation zone identification procedure.

The identification of material degradation bases on the previous identification of vibration mode shapes performed using CNN. The already-identified mode shapes are further investigated, which leads to the identification of the occurrence of the material degradation in the tested structure. If no degradation of the material is detected, the procedure ends. Otherwise, the identification of the degradation zone location and size is performed [36,63]. The whole procedure is shown in Figure 4 and can be described using three major steps:

1. Identification of mode shapes of the structure under the study.
2. Detection of the appearance of material degradation.
3. Identification of the size and location of material degradation zone.

This paper focuses on step 3, marked in Figure 4 with a yellow background. Steps 1 and 2 have been addressed in the previous paper by the same authors (see [36]); the accuracy of detection of the appearance of material degradation exceeding 99.8% (see Table 3) allows treating this step as practically error-free.

One should notice that the mode shapes are reduced before feeding them into the network. The original mode shape has a dimension of 6×80×121, which means that there are 6 degrees of freedom at each of the 80 nodes on each of the 121 rings distributed evenly along the length of the cylinder. The reduced mode shape has a dimension of 3×20×4: 3 displacements (rotational degrees of freedom are discarded) in 20 nodes (every fourth of the original 80) on four rings (see Figure 1a for rings A, B, C, and D in every fourth of the cylinder length).

### 3.3. Identification of the Zone of Material Degradation

Figure 4 shows the scheme of the material degradation zone identification procedure. The number of involved mode shapes creating the CNN input matrix is denoted by *i*. The values of *i* change from i=1 to i=6 in what follows, which means that from among all available mode shapes, at most, six are selected to form the CNN input matrix. The overall number of identified mode shapes equals 22; among them, the majority of mode shapes appear twice, with the same or almost the same natural frequency $f_{i}$ value. Those mode shapes are called *double* ones, and the others are called *single* ones. The following mode shapes are identified: A01 (axial mode), B11${}^{1}$, B11${}^{2}$ and B12${}^{1}$, B12${}^{2}$ (bending modes), C21${}^{1}$, C21${}^{2}$, C22${}^{1}$, C22${}^{2}$, C23${}^{1}$, C23${}^{2}$, C31${}^{1}$, C31${}^{2}$, C32${}^{1}$, C32${}^{2}$, C33${}^{1}$, C33${}^{2}$, C41${}^{1}$, C41${}^{2}$ and C42${}^{1}$, C42${}^{2}$ (circumferential modes), and T01 (torsional mode). Among the identified modes, the only single modes are axial and torsional (A01 and T01); from each pair of double modes, only the first mode is taken into account, and the second one is neglected; this leads to 12 modes under consideration: A01, B11${}^{1}$, B12${}^{1}$, C21${}^{1}$, C22${}^{1}$, C23${}^{1}$, C31${}^{1}$, C32${}^{1}$, C33${}^{1}$, C41${}^{1}$, C42${}^{1}$, and T01. In what follows, the superscript $\cdot^{1}$ is omitted for the simplicity of the description. The following error measures are used to present the results and discuss their accuracy:

- Mean Absolute Error (MAE):
  (5) $$\mathrm{MAE}=\frac{1}{n}\sum_{i=1}^{n}\left|\varepsilon_{i}\right|\;\;,$$
  where *n* is the number of identification cases and $\varepsilon_{i}=y_{i}-{\hat{y}}_{i}$ is the *i*-th error of identification, $y_{i}$ is the obtained, and ${\hat{y}}_{i}$ is the real value of the parameter being identified,
- Root Mean Squared Error (RMSE), defined by the following formula:
  (6) $$\mathrm{RMSE}=\sqrt{\frac{1}{n}\sum_{i=1}^{n}\varepsilon_{i}^{2}}\;\;.$$

During the performed analyses, FE models with the number of composite layers equal to 4, 8, 16, and 32 were considered. For each number of layers, 1000 models were generated with a random location and size of degradation zone. The cases where the number of layers equaled 4, 8, and 32 were used for CNN learning; the case with 16 layers was a test one. The following results will only present the data from testing; the results from learning had so little error that they can be treated as error-free.

In the first approach, each of the 12 mode shapes is separately taken in turn as a source of data in the procedure of the identification of the material degradation zone (see Figure 2a). The obtained results of the degradation zone location and size identification are shown in Table 4.

The name of the network, following the N$a^{b}$ scheme, represents the number *a* of the mode shapes in the network input matrix and the number of the variant *b* with a different combination of the same number of *a* mode shapes in the network input matrix.

It is visible that some mode shapes carry much more information about the damaged zone than others. The first bending and circumferential modes (B11 and C21; see Figure 1b,c) seem to be the most important ones from the point of view of the accuracy of the identification. To improve the accuracy of the identification, extended CNN input definitions have been tested, with two or more mode shapes simultaneously creating the network input matrix (see Figure 2). The above-mentioned modes B11 and C21 are applied together and in different configurations with some other mode shapes.

The results of the approach with two mode shapes involved are gathered in Table 5.

Table 6 shows the accuracy of the identification obtained with three mode shapes as input. The results are significantly worse when no bending mode B11 or no circumferential modes C*xx* are applied.

Table 7 and Table 8 present the results obtained on the basis of four, five, and six mode shapes as input.

## 4. Discussion of Results

**Figure 5:**
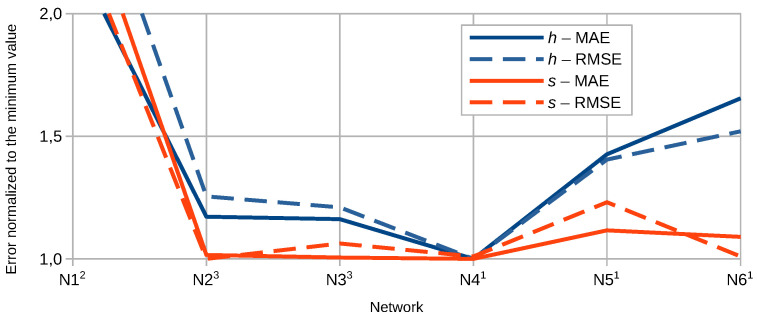
The results of the identification of location *h* and size *s* using different numbers of mode shapes.

To see the overall picture, the best results are summarized in Table 9. All the data presented in the table are also shown in Figure 5, where the error measures are normalized to their minimal values (1 means the highest accuracy among the obtained results of previous steps).

**Figure 6:**
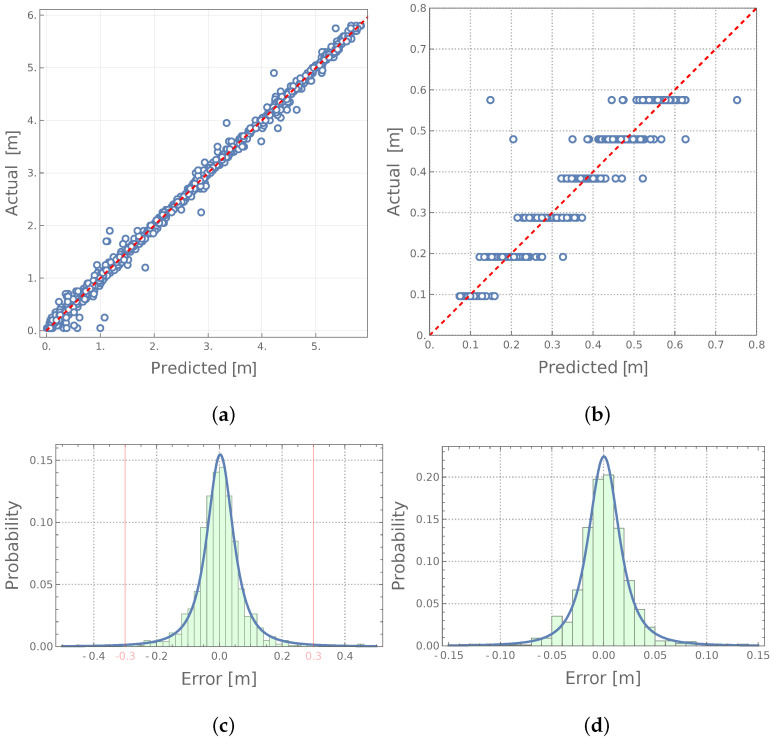
The results of identification of degradated zone: (**a**,**c**)—location; (**b**,**d**)—size.

The approach with four mode shapes on input, namely, two bending modes (B11, B12) and two circumferential modes (C21, C22), seems to give the most accurate results. The details of this approach are shown in Figure 6a,b in a form usually applied in the neural networks community (the horizontal axis presents the values identified by the network while the vertical axis shows the real, desired values). The closer the results are gathered around the diagonal x=y the better. However, this kind of diagram may be misleading in terms of the distribution of the results around the desired values; Figure 6c–d show the same data in the form of histograms together with the estimate of the error distribution. It is visible that the errors are concentrated around zero; in the case of the identification of the location of the degradation zone, the errors in the vast majority of cases do not exceed 0.30 m (note the vertical red lines in Figure 6d).

The identification efficiency is also evaluated by the Success Ratio (SR); see [64]:

(7) $$\mathrm{SR}=\frac{NbRe}{n}\times 100\%$$

where NbRe is the number of cases with the relative error not exceeding a particular restrained error $R_{e}$ (threshold) value $|\varepsilon_{i}/{\hat{y}}_{i}\times 100\%|\leq R_{e}$, and *n* is the number of all considered cases. The success ratio curve corresponds to the cumulative curve used in statistics.

**Figure 7:**
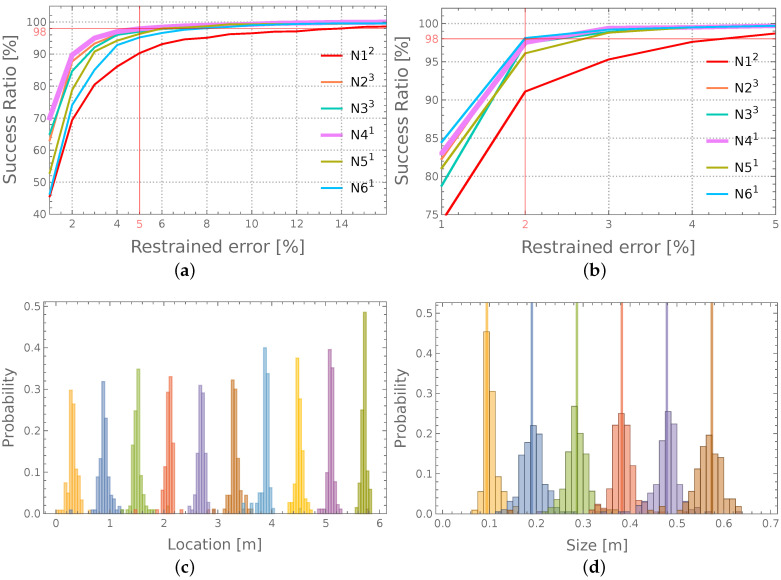
The histograms of errors of identification of degradated zone: (**a**,**c**)—location; (**b**,**d**)—size.

Figure 7a,b show the SR curves for location and size identification, respectively. In the case of the identification of the size of the degradation zone, the number of cases with an error lower than 2% exceeds 98% of all the considered cases; notice the horizontal red line in Figure 7 (N4${}^{1}$ network). In the case of the identification of the location, the same level of confidence (98% of all the considered cases) is reached with the relative error not exceeding 5% (N4${}^{1}$ network).

The data presented in the histogram in Figure 6d are also presented in a different form in Figure 7d; the difference is that all the errors presented collectively in Figure 6d are in Figure 7d divided into six subsets according to the real value of *s* (the size of the zone of material degradation). Each of these six sets is presented as a separate histogram, with its center placed on the horizontal axis at the location corresponding to the actual size of the identified zone. The areas where adjacent histograms overlap correspond to those cases where the error in size identification is large enough to cause the identified zone to be erroneously included in the adjacent set. The overlapping of the histograms has a very limited range; in the vast majority of cases, the classification (e.g., by choosing the closest possible answer) would give the correct value of parameter *s*.

Figure 7c shows similar histograms for the identification of the degradation zone location *h*. However, there is one major difference between Figure 7c and Figure 7d: the real values of *h* are almost continuous (h∈{0.1,5.95} m with the step of 0.05 m), and the ten set division is imposed when interpreting the results. The width of each set equals the length of twelve finite elements (0.6 m); the errors are calculated as a difference between the real and the identified value of *h*, even when the real value is different than the middle point of the particular set.

The approach presented in this paper—allowing to identify only one parameter describing the size of the material degradation zone (namely, the length of the side of the square degradation zone)—is a simplification of a real problem. In practice, the degradation zone may have different shapes and comprise a different number of composite material layers; a precise description of such damage obviously requires more parameters than one. However, the main problem analyzed in this paper was the usefulness of the method based on the identified vibration mode shapes for the localization of small damages of a size not exceeding 1.5% of the lateral surface of the analyzed structure. High accuracy of the approach proposed in the paper allows us to assume that it will also be possible to identify a larger number of parameters describing the damage, especially in the case of damage of larger sizes.

Experimental verification of the described method, for which the authors are preparing, would require vibration measurements (using, e.g., fast-camera image measurements with the DIC approach or laser vibrometer measurements) of the analyzed structure with the introduced damage and the application of modal analysis to determine the free vibration mode shapes. In the next step, the determined mode shapes should be identified (as described by the same authors in [36]), and finally, damage identification should be carried out using the procedure described in this article. It will require solving new problems, absent in the analysis of numerical data, including testing the resistance of the method to phenomena such as measurement noise and inaccuracies, or material non-homogeneity.

The accuracy of the damage zone identification should be assessed in the test equipment applied to precisely verify the identification results. In this paper, the accuracy of identification is evaluated in the context of using the measurement equipment at the authors’ disposal, namely, a mobile inspection system called C-CheckIR by Automation Technology^®^. The system may be applied for the detection of delaminations, water inclusions, debondings, etc., in composite materials. The area that can be examined in one position of the system is not smaller than about 30 × 30 cm, so the error in identifying a damaged zone location not exceeding 30 cm (5% of the highest possible value of *h*) enables the precise location of the damaged zone to be found at the first location of C-CheckIR measurement system.

## 5. Conclusions

The research results presented in this paper prove that it is possible to identify the location and size of a zone with degradation of the material properties solely based on carefully selected vibration mode shapes. The herein-established procedure only uses the mode shapes (displacements), does not use the natural frequencies or changes of these frequencies, and does not require information about the original (undamaged) state of the structure. The application of four mode shapes, the first two bending modes and the first two circumferential modes, are enough to obtain very high identification accuracy. The errors in identifying the degradation zone location *h* in 98% of considered cases does not exceed 5% of the length of the whole structure (for the cylinder with a length of 6 m, the errors are below 30 cm); in case of the size *s*, in 98% of cases the errors are lower than 3% (less than 2.8 cm). The reduction in the number of mode shapes (only the first bending mode shape and the first circumferential mode) results in a reduction in accuracy, but the identification errors are still very small.

It is of great importance that the accuracy does not depend on the location, i.e., the degradation zones located close to the fixed end of the cylinder are identified with similar accuracy to the ones located close to the free end of the cylinder.

## Figures and Tables

**Table 1:** Convolution segment architecture.

Layer Number	Input Type	Kernel Number	Kernel Size	Dimension of Data	Activation Function
1	Convolution	33	{2,5}	33 × 2 × 16	
2	Batch normalization			33 × 2 × 16	
3	Activation			33 × 2 × 16	ReLU
4	Convolution	66	{2,5}	66 × 1 × 12	
5	Batch normalization			66 × 1 × 12	
6	Activation			66 × 1 × 12	ReLU
7	Convolution	33	{1,3}	33 × 1 × 10	
8	Batch normalization			33 × 1 × 10	
9	Activation			33 × 1 × 10	ReLU
10	Pooling		{1,2}	33 × 1 × 5	
11	Flatten			165	

**Table 2:** The parameters *h* and *s* expressed in number of elements and in length units.

		*h*			*s*	
		NbFE	mm		NbFE	mm
Zone 1		6	300		4	191.6
Zone 2		18	900		8	383.2
Zone 3		34	1700		2	95.8
Zone 4		44	2200		12	574.8
Zone 5		64	3200		6	287.4
Zone 6		78	3900		10	479.0
Zone 7		96	4800		12	574.8
Zone 8		118	5900		2	95.8

**Table 3:** CNN accuracy of the identification of the material degradation occurence (for details, see [36]).

	Degradation Found	No Degradation Found	
Degradation exists in the model	1992	8	2000
No degradation exists	0	4000	4000
	1992	4008	

**Table 4:** The results of the identification of {h,s} on the basis of single mode shape.

Network Name	Mode Shape	*h*—Location		*s*—Size	
		MAE	RMSE	MAE	RMSE
		(m)	(m)	(m)	(m)
N1${}^{1}$	(A01)	1.4440	1.6800	0.1265	0.1492
N1${}^{2}$	(B11)	**0.1364**	**0.2947**	0.0489	0.0718
N1${}^{3}$	(B12)	0.3984	0.7122	0.0741	0.1015
N1${}^{4}$	(C21)	0.3238	0.4930	**0.0304**	**0.0480**
N1${}^{5}$	(C22)	0.3371	0.6896	0.0561	0.0831
N1${}^{6}$	(C23)	0.4642	0.7736	0.0753	0.1006
N1${}^{7}$	(C31)	0.5603	0.8180	0.0651	0.0931
N1${}^{8}$	(C32)	0.4024	0.7429	0.0634	0.0938
N1${}^{9}$	(C33)	0.4828	0.8146	0.0667	0.0972
N1${}^{10}$	(C41)	0.7011	1.0966	0.1044	0.1408
N1${}^{11}$	(C42)	0.8544	1.3096	0.1066	0.1402
N1${}^{12}$	(T01)	0.4244	0.7306	0.0844	0.1114

**Table 5:** The results of the identification of {h,s} on the basis of two mode shapes.

Network Name	Mode Shapes	*h*—Location		*s*—Size	
		MAE	RMSE	MAE	RMSE
		(m)	(m)	(m)	(m)
N2${}^{1}$	(B11,T01)	0.1195	0.2059	0.0468	0.0694
N2${}^{2}$	(B11,B12)	0.1315	0.2211	0.0478	0.0693
N2${}^{3}$	(B11,C21)	**0.0709**	**0.1320**	**0.0192**	**0.0303**
N2${}^{4}$	(B11,C22)	0.1578	0.2829	0.0411	0.0591
N2${}^{5}$	(B11,C31)	0.1126	0.1932	0.0331	0.0501
N2${}^{6}$	(B12,C21)	0.2298	0.4051	0.0271	0.0438
N2${}^{7}$	(C21,C31)	0.3018	0.4790	0.0253	0.0371

**Table 6:** The results of the identification of {h,s} on the basis of three mode shapes.

Network Name	Mode Shapes	*h*—Location		*s*—Size	
		MAE	RMSE	MAE	RMSE
		(m)	(m)	(m)	(m)
N3${}^{1}$	(B11,B12,T01)	0.1503	0.2575	0.0521	0.0754
N3${}^{2}$	(B11,B12,C21)	0.0831	0.1517	0.0212	0.0393
N3${}^{3}$	(B11,C21,C22)	**0.0703**	**0.1273**	**0.0190**	**0.0322**
N3${}^{4}$	(B11,C21,C31)	0.1242	0.2242	0.0222	0.0384
N3${}^{5}$	(C21,C31,C41)	0.4245	0.6697	0.0267	0.0371
N3${}^{6}$	(B11,C21,T01)	0.0955	0.1748	0.0204	0.0385

**Table 7:** The results of the identification of {h,s} on the basis of four mode shapes.

Network Name	Mode Shapes	*h*—Location		*s*—Size	
		MAE	RMSE	MAE	RMSE
		(m)	(m)	(m)	(m)
N4${}^{1}$	(B11,B12,C21,C22)	**0.0605**	**0.1052**	**0.0189**	**0.0306**
N4${}^{2}$	(B11,C21,C22,C32)	0.1131	0.2175	0.0219	0.0414
N4${}^{3}$	(B11,C21,C31,C41)	0.1642	0.2744	0.0229	0.0367
N4${}^{4}$	(B11,C21,C31,T01)	0.1290	0.2239	0.0196	0.0324

**Table 8:** The results of the identification of {h,s} on the basis of five or six mode shapes.

Network Name	Mode Shapes	*h*—Location		*s*—Size	
		MAE	RMSE	MAE	RMSE
		(m)	(m)	(m)	(m)
N5${}^{1}$	(B11,B12,C21,C22,T01)	**0.0863**	**0.1478**	**0.0211**	**0.0373**
N5${}^{2}$	(B11,C21,C31,C41,T01)	0.1043	0.1651	0.0226	0.0384
N6${}^{1}$	(B11,B12,C21,C22,C31,C32)	0.1001	0.1599	0.0206	0.0306
N6${}^{2}$	(B11,B12,C21,C22,C31,T01)	0.1004	0.1789	0.0201	0.0338

**Table 9:** The best results of the identification of {h,s}.

Network Name	Mode Shape	*h*—Location		*s*—Size	
		MAE	RMSE	MAE	RMSE
		(m)	(m)	(m)	(m)
N1${}^{2}$	(B11)	0.1364	0.2947	0.0489	0.0718
N2${}^{3}$	(B11,C21)	0.0709	0.1320	0.0192	0.0303
N3${}^{3}$	(B11,C21,C22)	0.0703	0.1273	0.0190	0.0322
**N4** ${}^{1}$	**(B11,B12,C21,C22)**	**0.0605**	**0.1052**	**0.0189**	**0.0306**
N5${}^{1}$	(B11,B12,C21,C22,T01)	0.0863	0.1478	0.0211	0.0373
N6${}^{1}$	(B11,B12,C21,C22,C31,C32)	0.1001	0.1599	0.0206	0.0306

## Data Availability

The data underlying this article will be shared by the corresponding author upon reasonable request.

## Abbreviations

The following abbreviations are used in this manuscript:

BP	Back-propagation
CNN	Convolutional Neural Network
DIC	Digital Image Correlation
FE	Finite Element
FEM	Finite Element Method
MAE	Mean Average Error
MLP	Multi-Layer Perceptron
NbFE	Number of Finite Elements
NDT	Non-destructive Testing
ReLU	Rectified Linear Units
RMSE	Root Mean Squared Error
SHM	Structural Health Monitoring
SR	Success Ratio
SVM	Support Vector Machine

## References

[1] Irving P.E., Soutis C. (2014). Polymer Composites in the Aerospace Industry.

[2] Jawaid M., Thariq M. (2018). Sustainable Composites for Aerospace Applications.

[3] Katunin A. (2019). Aircraft Structures: Mechanics, Design, and Maintenance.

[4] Hollaway L. (1993). Polymer Composites in the Aerospace Industry.

[5] Uddin N. (2013). Developments in Fiber-Reinforced Polymer (FRP) Composites for Civil Engineering.

[6] ACT of 3 July 2002 Aviation Law (Poland). Dz.U. 2020 r. 2020, poz. 1970. Consolidated Text. https://ec.europa.eu/growth/tools-databases/regprof/index.cfm?action=regprof&id_regprof=13006.

[7] Janas L., Miller B., Kaszyński A. (2018). Computational algorithms supporting the bridge management system. Balt. J. Road Bridge Eng..

[8] Karbhari V.M., Ansari F. (2005). Health Monitoring, Damage Prognosis and Service-Life Prediction—Issues Related to Implementation.

[9] Moore P.O. (2010). Nondestructive Testing Handbook, Volume 9, Visual Testing (VT).

[10] Gholizadeh S. (2016). A review of non-destructive testing methods of composite materials. Procedia Struct. Integr..

[11] Bossi R.H., Giurgiutiu V., Irving P.E., Soutis C. (2014). Chapter nondestructive testing of damage in aerospace composites. Polymer Composites in the Aerospace Industry.

[12] Fotouhi S., Pashmforoush F., Bodaghi M., Fotouhi M. (2021). Autonomous damage recognition in visual inspection of laminated composite structures using deep learning. Compos. Struct..

[13] Ensminger D., Bond L.J. (2011). Ultrasonics: Fundamentals, Technologies, and Applications.

[14] Scott I., Scala C. (1982). A review of non-destructive testing of composite materials. NDT Int..

[15] Holland S.D., Reusser R.S. (2016). Material Evaluation by Infrared Thermography. Annu. Rev. Mater. Res..

[16] Sikdar S., Kundu A., Jurek M., Ostachowicz W. (2019). Nondestructive Analysis of Debonds in a Composite Structure under Variable Temperature Conditions. Sensors.

[17] Crane R.L., Beaumont P.W.R., Zweben C.H. (2018). 7.10 Radiographic Inspection of Composite Materials. Comprehensive Composite Materials II.

[18] Wu R., Zhang H., Yang R., Chen W., Chen G. (2021). Nondestructive Testing for Corrosion Evaluation of Metal under Coating. J. Sens..

[19] Kant R., Chauhan P.S., Bhatt G., Bhattacharya S., Bhattacharya S., Agarwal A.K., Prakash O., Singh S. (2019). Corrosion Monitoring and Control in Aircraft: A Review. Sensors for Automotive and Aerospace Applications.

[20] Jurek M., Radzienski M., Kudela P., Ostachowicz W. (2018). Non-contact excitation and focusing of guided waves in CFRP composite plate by air-coupled transducers for application in damage detection. Procedia Struct. Integr..

[21] Katunin A. (2018). A Concept of Thermographic Method for Non-Destructive Testing of Polymeric Composite Structures Using Self-Heating Effect. Sensors.

[22] Hou R., Xia Y. (2021). Review on the new development of vibration-based damage identification for civil engineering structures: 2010–2019. J. Sound Vib..

[23] Fan W., Qiao P. (2011). Vibration-based Damage Identification Methods: A Review and Comparative Study. Struct. Health Monit..

[24] Cao M.S., Sha G.G., Gao Y.F., Ostachowicz W. (2017). Structural damage identification using damping: A compendium of uses and features. Smart Mater. Struct..

[25] Farrar C., James G. (1997). System Identification from Ambient Vibration Measutements on a Bridge. J. Sound Vib..

[26] Dessi D., Camerlengo G. (2015). Damage identification techniques via modal curvature analysis: Overview and comparison. Mech. Syst. Signal Process..

[27] Zhu J. (2021). Review on Structural Health Monitoring of Offshore Platform. J. Phys..

[28] Pooya S.M.H., Massumi A. (2021). A novel and efficient method for damage detection in beam-like structures solely based on damaged structure data and using mode shape curvature estimation. Appl. Math. Model..

[29] Hadjileontiadis L., Douka E., Trochidis A. (2005). Fractal dimension analysis for crack identification in beam structures. Mech. Syst. Signal Process..

[30] Liew K.M., Wang Q. (1998). Application of Wavelet Theory for Crack Identification in Structures. J. Eng. Mech..

[31] Huang N.E., Attoh-Okine N.O. (2005). The Hilbert-Huang Transform in Engineering.

[32] Wang W., Mottershead J.E., Mares C. (2009). Mode-shape recognition and finite element model updating using the Zernike moment descriptor. Mech. Syst. Signal Process..

[33] Zang C., Lan H.B., Jiang D.D., Friswell M.I. (2021). Mode Shape Description and Model Updating of Axisymmetric Structures Using Radial Tchebichef Moment Descriptors. Shock Vib..

[34] Miller B., Ziemiański L. (2020). Optimization of dynamic behavior of thin-walled laminated cylindrical shells by genetic algorithms and deep neural networks supported by modal shape identification. Adv. Eng. Softw..

[35] Miller B., Ziemiański L. (2020). Optimization of Dynamic and Buckling Behavior of Thin-Walled Composite Cylinder, Supported by Nature-Inspired Agorithms. Materials.

[36] Miller B., Ziemiański L. (2021). Identification of Mode Shapes of a Composite Cylinder Using Convolutional Neural Networks. Materials.

[37] Wang W., Mottershead J.E., Ihle A., Siebert T., Reinhard Schubach H. (2011). Finite element model updating from full-field vibration measurement using digital image correlation. J. Sound Vib..

[38] Molina-Viedma Á.J., López-Alba E., Felipe-Sesé L., Díaz F.A. (2017). Full-field modal analysis during base motion excitation using high-speed 3D digital image correlation. Meas. Sci. Technol..

[39] Stanbridge A.B., Martarelli M., Ewins D.J. (2004). Measuring area vibration mode shapes with a continuous-scan LDV. Measurement.

[40] Hinton G.E., Osindero S., Teh Y.W. (2006). A Fast Learning Algorithm for Deep Belief Nets. Neural Comput..

[41] LeCun Y., Bengio Y., Hinton G. (2015). Deep learning. Nature.

[42] Montavon G., Samek W., Müller K.R. (2018). Methods for interpreting and understanding deep neural networks. Digit. Signal Process..

[43] Goodfellow I., Bengio Y., Courville A. (2016). Deep Learning.

[44] Abdeljaber O., Avci O., Kiranyaz S., Gabbouj M., Inman D.J. (2017). Real-time vibration-based structural damage detection using one-dimensional convolutional neural networks. J. Sound Vib..

[45] Modarres C., Astorga N., Droguett E.L., Meruane V. (2018). Convolutional neural networks for automated damage recognition and damage type identification. Struct. Control. Health Monit..

[46] Tang Z., Chen Z., Bao Y., Li H. (2019). Convolutional neural network-based data anomaly detection method using multiple information for structural health monitoring. Struct. Control Health Monit..

[47] Zhong K., Teng S., Liu G., Chen G., Cui F. (2020). Structural Damage Features Extracted by Convolutional Neural Networks from Mode Shapes. Appl. Sci..

[48] Teng S., Chen G., Gong P., Liu G., Cui F. (2020). Structural damage detection using convolutional neural networks combining strain energy and dynamic response. Meccanica.

[49] Poggio T., Mhaskar H., Rosasco L., Miranda B., Liao Q. (2017). Why and when can deep-but not shallow-networks avoid the curse of dimensionality: A review. Int. J. Autom. Comput..

[50] Chen W., Shi K. (2019). A deep learning framework for time series classification using Relative Position Matrix and Convolutional Neural Network. Neurocomputing.

[51] Oishi A., Yagawa G. (2017). Computational mechanics enhanced by deep learning. Comput. Methods Appl. Mech. Eng..

[52] Deng H., Zhang W.x., Liang Z.f. (2021). Application of BP Neural Network and Convolutional Neural Network (CNN) in Bearing Fault Diagnosis. IOP Conf. Ser. Mater. Sci. Eng..

[53] Hasan M., Ullah S., Khan M.J., Khurshid K. (2019). Comparative Analysis of SVM, ANN and CNN for Classifying Vegetation Species Using Hyperspectral Thermal Infrared Data. Int. Arch. Photogramm. Remote. Sens. Spat. Inf. Sci..

[54] Liu W., Wei J., Meng Q. Comparisions on KNN, SVM, BP and the CNN for Handwritten Digit Recognition. Proceedings of the 2020 IEEE International Conference on Advances in Electrical Engineering and Computer Applications (AEECA).

[55] Lee B., Kam D., Cho Y., Kim D.C., Lee D.H. (2021). Comparing Performances of CNN, BP, and SVM Algorithms for Differentiating Sweet Pepper Parts for Harvest Automation. Appl. Sci..

[56] Clough R.W., Penzien J. (2003). Dynamics of Structures.

[57] Bathe K. (1996). Finite Element Procedures.

[58] Vo T.P., Lee J., Ahn N. (2009). On sixfold coupled vibrations of thin-walled composite box beams. Compos. Struct..

[59] Bathe K. (2016). ADINA: Theory and Modeling Guide Volume I: ADINA Solids & Structures.

[60] Miller B., Ziemiański L., Pietraszkiewicz W., Witkowski W. (2018). Chapter Numerical analysis of free vibrations of a tube shaped laminated cantilever. Proceedings of the 11th International Conference Shell Structures: Theory and Applications, (SSTA 2017).

[61] Miller B., Ziemiański L. (2019). Maximization of Eigenfrequency Gaps in a Composite Cylindrical Shell Using Genetic Algorithms and Neural Networks. Appl. Sci..

[62] (2019). Mathematica, Version 12.

[63] Bunting G., Miller S.T., Walsh T.F., Dohrmann C.R., Aquino W. (2021). Novel strategies for modal-based structural material identification. Mech. Syst. Signal Process..

[64] Kuźniar K., Waszczyszyn Z., Lagaros N., Tsompanakis Y. (2007). Chapter Neural Networks for the Simulation and Identification Analysis of Buildings Subjected to Paraseismic Excitations. Intelligent Computational Paradigms in Earthquake Engineering.

